# Experiences of patients identifying with chronic Lyme disease in the healthcare system: a qualitative study

**DOI:** 10.1186/1471-2296-15-79

**Published:** 2014-05-01

**Authors:** Ather Ali, Lawrence Vitulano, Robert Lee, Theresa R Weiss, Eve R Colson

**Affiliations:** 1 Department of Pediatrics, Yale School of Medicine, P.O. Box 208064, New Haven, CT 06520-8064, USA; 2 Child Study Center, Yale School of Medicine, P.O. Box 208064, New Haven, CT 06520-8064, USA

**Keywords:** Chronic Lyme, Health beliefs, Burden, Complementary medicine, Qualitative, Lived experiences

## Abstract

**Background:**

Chronic Lyme disease is a term that describes a constellation of persistent symptoms in patients with or without evidence of previous *Borrelia burgdorferi* infection. Patients labeled as having chronic Lyme disease have a substantial clinical burden. Little is known about chronic Lyme disease patient experiences in the healthcare system and their relationships with healthcare providers. The purpose of this study was to gather insights about the experiences of patients who carry a diagnosis of chronic Lyme disease in the United States healthcare system.

**Methods:**

Qualitative, phenomenological study in 12 adult participants who identified themselves as having chronic Lyme disease. Semi-structured face-to-face in-depth interviews were conducted, 60–90 minutes in length, focusing on perceptions of disease burden and of their healthcare providers, using the dimensions of the Health Belief Model. Transcribed interviews were analyzed for emergent topics and themes in the categories of beliefs/understanding, personal history/narrative, consequences/limitations, management, and influences on care.

**Results:**

Enrollment continued until theoretical saturation was obtained. Four major themes emerged from participants’ descriptions of their experiences and perceptions: 1) changes in health status and the social impact of chronic Lyme disease, 2) doubts about recovery and the future, 3) contrasting doctor-patient relationships, 4) and the use of unconventional therapies to treat chronic Lyme disease.

**Conclusions:**

Participants reported a significant decline in health status associated with chronic Lyme disease and were often unsatisfied with care in conventional settings. Negative experiences were associated with reports of dismissive, patronizing, and condescending attitudes. Positive experiences were associated with providers who were reported to be attentive, optimistic, and supportive. Consultations with CAM practitioners and use of CAM therapies were common. Actively engaged and sympathetic clinical encounters may foster greater satisfaction in healthcare settings.

## Background

Chronic Lyme disease (CLD) is a term that describes a constellation of persistent symptoms in patients with or without exposure to *Borrelia burgdorferi*. These symptoms may include fatigue, night sweats, arthralgia, myalgia, arrhythmias, abdominal pain, nausea, diarrhea, sleep disturbance, cognitive dysfunction, irritability, depression, back pain, and headache [1]. There is no standard definition of CLD; the Infectious Diseases Society of America distinguishes between ‘post-Lyme disease syndrome’ or ‘late Lyme disease’ in which arthralgia and other symptoms persist after documented *B. burgdorferi* infection [2], while Feder et al. [1] categorize similar syndromes into post-Lyme disease syndrome, symptoms of unknown cause (with our without antibodies to *B. burgdorferi),* and defined illnesses unrelated to *B. burgdorferi* infection.

Nevertheless, patients are often are diagnosed with CLD based on nonstandard interpretations of serology or other testing that has little or no validity, or based on clinical symptoms alone [2,3]. CLD is diagnosed throughout the U.S. and globally, in both endemic and non-endemic areas [1]. Individuals may make the diagnosis themselves using lists of subjective symptoms [4,5]. Treatment protocols can include regimens of multiple antibiotics (oral or parenteral), sometimes continuing for months or years [2,3]. These protocols are associated with substantial risks such as out-of-pocket expenses, patient distress, potential harm, as well as increasing risk of selecting for antibiotic resistant bacteria [1,6].

One survey found that 2.1% of Connecticut-based primary care physicians diagnose and treat CLD, while the majority were unsure of or did not believe in CLD [7]. The predominant infectious diseases, pediatric, and neurology academic societies do not recognize CLD as a distinct clinical entity, while other professional and advocacy organizations argue for a broader definition of CLD focusing on clinical (non-serological) diagnosis based on reported symptomology [3,8]. Patient perceptions of lack of sympathy and alienation from mainstream medical institutions led to the growth of advocacy groups supporting long-term antibiotic treatment, as well as fostering ‘us vs. them’ sentiments in the advocacy groups [8].

Qualitative methods are well suited to generate rich data about phenomena in their context and can help develop hypotheses for further research, as well as inform the development of patient-centered interventions [9]. The purpose of this study was to gather insights about the experiences of patients identifying themselves as having CLD in the healthcare system using the dimensions of the Health Belief Model where perceived severity, susceptibility, benefits, and barriers; as well as cues to action, and self-efficacy are involved in influencing health behavior choices [10]. We also aimed to gain insights into therapeutic and healthcare provider choices to better understand their illness representations, that is, their beliefs and expectations about an illness [11,12].

## Methods

### Study design and sample

This was a qualitative, descriptive study in which face-to-face in-depth interviews were conducted with patients who were diagnosed with or self-identify as having CLD. Purposive sampling [13] was used to recruit participants from Connecticut-based patient-oriented Lyme disease email lists and the website, craigslist.org. Recruitment announcements solicited participants with CLD (diagnosed by a clinician or by self-diagnosis) and were willing to complete an in-person interview. Participants were not known to the investigators prior the study.

Enrollment continued until theoretical saturation was obtained; i.e., the point at which no new concepts emerged in a category, and in which categories were well characterized and differentiated [9]. Demographic data included sex, age, race/ethnicity (NIH criteria), years of education, religious preference, marital status, number of children/dependents, employment, and health insurance status. Subjects were told that the aim of the study was to gain insights into the experiences of patients with CLD and that this information will be used to develop interventions to improve patient care and satisfaction.

### Ethics statement

The study was approved by the Human Investigation Committee (HIC) at the Yale School of Medicine and was compliant with HIPAA regulations.

### Strategy of inquiry

An interview guide was created based on the Health Belief Model [10] dimensions of perceived susceptibility, severity, benefits, and barriers, as well as mediating factors such as cues to action and self-efficacy (see Table 1). The goals of the interview were not only to gather information about what choices were made, but to gain and understanding of the decision-making process of patients with CLD. We were also interested in assessing the rationale of provider and therapeutic choice, and specific factors patients felt were important in their decision-making processes.

**Table 1:** Interview guide

**Health belief model construct/mediating factors**	**Question(s)**
*Perceived Susceptibility*	How would you describe your health prior to CLD?
	Is there anything that you could have done differently to avoid getting CLD?
	Is there anything that people can do to avoid getting CLD?
*Perceived Severity*	How did you feel when you were diagnosed with (or discovered that you had) CLD?
	How has CLD affected your life?
	What does having CLD mean to you?
*Perceived Benefits*	What treatments have you had for CLD?
	How have these treatments affected you?
*Perceived Barriers*	Have there been any obstacles that you have encountered in getting treatment for CLD?
	What needs to be done to restore you to optimum health?
	Do you anticipate that you’ll fully recover from CLD?
	What have the doctors that have least helped your CLD not done for you?
*Cues to Action*	What kinds of support (community, family, friends, health care providers, CLD support groups, internet newsgroups, CLD activism groups, etc.) have you had during your CLD?
	How have these support systems affected your CLD?
	What motivates you in your struggle with CLD?
*Self Efficacy*	What can doctors do to more effectively care for patients with CLD?
	What can the community do to best support people with CLD?
*CLD Diagnosis and Healthcare System*	Why does CLD best fit your symptoms?
	Have you ever doubted that you have CLD?
	How do you think conventional health care providers view CLD patients?
	How do you think LLMDs view CLD patients?
	What have the doctors that have helped your CLD the most done for you?

CLD = Chronic Lyme disease.

LLMD = Lyme literate medical doctors.

Interviews were face-to-face, semi-structured and focused on the perceptions and experiences of patients, including health beliefs, diagnostic methods, therapeutic regimens, and perceived efficacy, using the dimensions of the Health Belief Model [10]. Prior to each interview, written informed consent was obtained and participants rated their likelihood of recovery on a visual analog scale and sense of overall well-being.

Interviews took place either at Yale University (New Haven, CT) or at participants’ place of residence, lasted between 60–90 min, and were conducted by two investigators (AA; CAM-trained research scientist and a medical student, both male). Interviews were recorded digitally and transcribed by a HIPAA-compliant service (Transcription Plus LLC, Bristol, CT).

### Data analysis

Interviews were conducted with twelve participants; one additional interview was conducted to corroborate findings and assess saturation. Using a hermeneutic, phenomenological methodology, the focus of inquiry was placed on the patients’ lived experience with chronic Lyme disease [14]. Transcripts were analyzed using standard methods of content analysis [9,13]. After completing the first three interviews, transcripts were read by the multidisciplinary investigative team for an overall understanding to identify emergent themes. After subsequent interviews, and during the iterative process of reviewing each transcript as it is collected, the list of themes was revised multiple times. Codes were assigned to specific statements in each transcript, in five categories: 1) beliefs/understanding, 2) personal history/narrative, 3) consequences/limitations, 4) management, and 5) influences on care. Themes were then condensed from these categories and codes. Data from all of the transcripts was coded using ATLAS.ti 6.1 (ATLAS.ti Scientific Software Development GmbH, Berlin) by one investigator (AA). Participants were not involved in the data analysis and interpretation.

## Results

### Participants

All participants were White with a mean age of 41 years (range 21–69). Seven participants were college graduates, and eleven participants had health insurance. Of the eleven insured, CLD treatments were partially or fully covered for eight participants (see Table 2).

**Table 2:** Demographic and social characteristics of participants

**Participant**	**Age**	**Gender**	**Marital status**	**Employment**	**CLD diagnosis**	**Health insurance status**	**CLD treatment insurance coverage**	Perceived wellbeing (24-hr)*	Perceived wellbeing (1-month)*	Perceived likelihood of recovery**
1	55	F	Married	Disability	Provider + Self	Insured	Partial	57	45	13
2	51	M	Married	Full-time	Provider	Insured	Partial	65	75	51
3	25	F	Married	Unemployed	Provider	Uninsured	-	62	53	73
4	53	F	Single	Part-time	Self	Insured	No	69	71	77
5	23	F	Married	Full-time	Provider	Insured	No	42	23	27
6	21	F	Single	Full-time	Provider	Insured	No	63	59	51
7	49	M	Single	Unemployed	Provider	Insured	Partial	82	64	65
8	45	F	Single	Part-time	Self	Insured	Full	44	56	67
9	47	F	Civil Union	Full-time	Provider + Self	Insured	Partial	73	65	91
10	31	M	Separated	Unemployed	Provider + Self	Insured	Partial	38	27	10
11	28	F	Single	Full-time	Provider + Self	Insured	Partial	87	75	85
12	69	F	Single	Full-time	Provider + Self	Insured	Full	80	61	0

*The Arizona Integrative Outcomes scale assesses a global sense of spiritual, social, mental, emotional, and physical wellbeing and is anchored by the statements 0 = "worst you have ever been" and 100 = "best you have ever been" [15].

**100 mm Visual Analog Scale anchored by the statements 0 = "will never recover" and 100 = "will recover completely".

### Sense of well-being and likelihood of recovery

Prior to each interview, participants completed the Arizona Integrative Outcomes Scale [15], assessing a global sense of spiritual, social, mental, emotional, and physical well-being, anchored by the opposing statements, "worst you have ever been (0)" and "best you have ever been (100)" [15] on a 10-cm visual analog scale. Mean scores were 64 mm (range 42–87) for the 24-hour assessment, and 56 mm (range 27–75) for the previous month. Participants were also asked about their likelihood of recovery from CLD using a 10-cm visual analog scale anchored by "will never recover (0)" to "will recover completely (100)." Mean scores were 31 (range 0–91).

### Themes

We discussed with each participant their perceptions of susceptibility to CLD, severity of symptoms, benefits of treatment, and barriers to treatment and recovery using the dimensions of the Health Belief Model [10]. We also queried cues to action and self-efficacy in terms of external support and motivation, as well as questions about the diagnostic label of CLD and experiences in the healthcare system in terms of physician interaction and financial aspects (See Table 1).

Four major themes emerged from participants’ descriptions of their experiences and perceptions. Patients discussed: 1) Changes in health status and social impact of chronic Lyme disease, 2) Doubts about recovery and the future, 3) Contrasting doctor-patient relationships, and 4) Unconventional therapies to treat chronic Lyme disease.

#### Theme 1: Changes in health status and social impact of chronic Lyme disease

Eleven of the twelve participants noted that they were highly functional in their daily lives prior to CLD and described themselves as "athletic," "healthy," "perfect," "never sick before," and "energetic." All expressed substantial degradations in their quality of life after acquiring CLD. One expressed frustration with the changes to her daily routine:

"I was in excellent health before …and that’s what really upsets me the most is the Lyme has taken away my job that I love, making me not be able to work and taken away my activities." (Participant #1)

Nine participants discussed how CLD symptoms adversely affected their daily activities, while nine participants reported professional limitations, including prolonged absences from work or school or the need to enroll in a disability program. Eight participants noted social limitations, including feeling isolated, not being understood, or others not believing that they were ill. One participant noted that fatigue associated with CLD resulted in social isolation:

" I don’t feel like getting myself all riled up and dressed up to go out on a social occasion. I’d rather just sit back and be at home… I certainly have noticed that I’ve become a different person since having Lyme disease." (Participant #8)

Eight participants noted out-of-pocket financial costs associated with CLD treatment; many spent thousands to tens of thousands of dollars on care. One participant discussed his costs in the context of insurance deductibles:

"Even though insurance pays for this… I have already reached my out-of-pocket max and it’s August. I met it last month and that’s $2500. That doesn’t include other prescriptions or co-pays for August visits. That’s just procedures like an MRI and this whole--everything to do with this--the treatment and the inserting the [PICC] line, and the weekly nurses." (Participant #11)

#### Theme 2: Doubts about recovery and the future

Six participants were unsure about whether they would ever fully recover from CLD, while three participants felt that they would fully recover at some point in the future. One participant stated that she was currently recovering from CLD. Perceived likelihood of recovery was generally related to beliefs surrounding the pathogenesis of CLD. One participant noted:

"I’ve heard that you never fully recover--that it goes into remission, so I’m not really sure if I do but I hope maybe I will be able to." (Participant #5)

One person expressed dismay upon seeing the extent of disability in support group participants.

"I went to the first Lyme support group meeting; there were people there with canes, people in wheelchairs and I said, ‘No way…not me.’ I was like, ‘I can’t do that, I can’t be that.’" (Participant #2)

In addition to concerns about persistent symptoms, six participants were fearful that they might die from CLD, while two discussed CLD-related suicidal thoughts. One participant noted that her symptoms were so debilitating that her will to live was broken:

"I got really, really depressed and I didn’t want to live…I ripped my PICC line out of my arm. I said, ‘I’m going to die a slow death.’ I was begging [my boyfriend] to smother me with a pillow." (Participant #10)

#### Theme 3: Contrasting doctor-patient relationships

All participants reported strong feelings about what constituted high quality care and characteristics of physicians that have been most helpful in treating CLD. Two divergent types of doctor-patient relationships emerged; physicians were characterized as either exceptionally supportive, or uncaring and dismissive.

Five participants characterized helpful doctors as attentive and good listeners. Other attributes describing helpful doctors included willingness to acknowledge patient concerns, open-mindedness, being supportive, optimism, and a holistic approach. One participant described a helpful physician as:

"They treat you like, like you’re someone in pain and you’re someone that’s worthy of getting better and getting treatment and that’s huge, ‘cause you don’t get that from a lot of people." (Participant #3)

Eleven participants discussed negative experiences with clinicians. Nine participants described some clinicians as dismissive of the diagnosis of CLD or the severity of their symptoms. Six participants described the ‘unhelpful doctors’ as condescending or patronizing. One participant felt that her doctor was unsupportive of her concerns regarding CLD as a cause of her symptoms:

"Walking in the door, he knew more than we did. Before asking us any questions at all, I felt he’d already made his mind up…When I asked, ‘Well what about Lyme?’- he never tested…never asked any questions. ‘It can’t be,’ [he said]. … It’s like, ‘Well who are we? We have no control over our lives? We’re stupid? We don’t know what’s going on that you wouldn’t even consider this?’" (Participant #4)

Another participant was frustrated with her primary care physician for not accepting her CLD status and sought out other providers.

"I couldn’t continue to see someone who didn’t believe I have a disease that I know I have. You know, it’s a chronic disease and I would see him every year for my annual exam and he didn’t believe me." (Participant #9)

#### Theme 4: Unconventional therapies to treat chronic Lyme disease

As most participants described some negative encounters with mainstream healthcare providers and ongoing symptomology, many sought out providers that supported prolonged antibiotic regimens (‘Lyme Literate Medical Doctors - LLMD’ [8]) or complementary and alternative medicine (CAM) therapies and providers.

All participants reported receiving multiple antibiotic regimens (oral and/or intravenous) lasting months or years, and all participants reported symptomatic relief while using the antibiotics. Despite reporting relief from conventional treatments (antibiotics) used for durations beyond standard guidelines [2], eleven of our twelve participants reported utilizing one or more types of CAM therapies and providers to treat CLD. Nine participants visited a naturopathic doctor (ND), while one participant reported consulting a chiropractor (DC). Reported CAM therapies are listed in Table 3.

**Table 3:** Reported use of complementary and alternative (CAM) therapies

**Vitamins and minerals**	Vitamin C
	Vitamin D
	Magnesium
	Colloidal silver
**Herbal medicines**	Astragalus
	Cat’s claw
	Garlic
	Mushroom extracts
	Wormwood
**Natural products**	Coenzyme Q10
	Probiotics
	Evening primrose oil
	Fish oil
	L-carnitine
**Other CAM modalities**	Acupuncture
	Reiki
	Rife machine
	Vega machine
**Unconventional laboratory testing**	Heavy metal serum levels

A number of participants visited CAM providers on referral from or in collaboration with their LLMDs [8] to address side effects of antibiotics, to treat other symptoms, to provide a holistic approach, or to consult a sympathetic clinician. One participant described her experience in treating leg pain as well as skepticism regarding unconventional treatments:

"I went to this Naturopath and she did some acupuncture and Reiki and I did feel this just amazing wave go through my legs and she felt it too… I said, ‘I feel it’s like this energy just shooting through,’ and she put this needle in my ear and I felt it go all the way down and out through my legs…like it released some blockage or something…I never put much credibility to that, so it gave me a new perspective." (Participant #2)

Other participants were drawn to a holistic approach from CAM providers. This 21-year old participant felt that CLD prematurely aged her, and that the CAM provider addressed this concern:

"He’s not just attacking the Lyme; he has to get our toxicity levels down before he even starts that. Right now I have the toxicity level of a healthy 65 year old. So he goes by steps, but he understands the whole aspect of Lyme disease, not just that you need to throw antibiotics at people…because otherwise it’s not going to do anything. So, just like he goes in steps and because it’s slow you don’t ‘hurx’ (Jarisch-Herxheimer reaction) as bad." (Participant #6)

## Discussion

Our participants reported substantial limitations in their daily lives and a decline in health status attributed to CLD. Some believed that they would never recover and may possibly die from CLD. Participants had polarized experiences in the healthcare system: negative experiences were associated with providers who were reported to be dismissive, patronizing, or condescending, while positive experiences were associated with providers reported to be optimistic, attentive, and supportive. Consultations with CAM practitioners and use of CAM therapies were common.

Our cohort included participants who were diagnosed with or self-identify as having CLD irrespective of whether they had objective evidence of previous borreliosis. Because our study aimed to better understand and describe individual and collective experiences and ideas (e.g., the perception of a disease), strict enrollment criteria utilizing serology would not be helpful. Therefore, the only criterion for enrollment was that a person identified as a CLD patient; the etiology and classification of their symptoms were not relevant.

Reported cases of Lyme disease to the Centers for Disease Control and Prevention (meeting surveillance criteria) are most common in the Northeastern United States among children aged 5–9, and adults aged 55–59, with male predominance (53%). Most reported cases (94%) occur in patients identified as White [16]. Our study focused on adults; 75% of our cohort were female, all were White.

Most participants reported general health and wellness prior to CLD. In previous literature, health-related quality of life among CLD patients has been reported to be similar to patients with congestive heart failure and type 2 diabetes [17]. Our participants also had a mean score of 56 on the Arizona Integrative Outcomes Scale (AIOS) for the month prior to their interview, indicating a compromised sense of well-being. Of note, Bell et al. reported similar mean AIOS scores of 51 for individuals who qualified as "psychologically distressed" on the Global Severity Index (a standard measure of psychological health) [15].

It is possible that this psychological distress associated with severe symptomology, coupled with a lack of clear disease course and uncertainty about recovery led some to fear dying from CLD, despite CDC data indicating that Lyme disease rarely causes death [18]. Previous studies have found an association between CLD and negative affect [19], and mixed results regarding an association with anxiety and/or depression [19,20].

Our findings also provide insight into the dimensions of Leventhal’s illness representations of identity, cause, timeline, control/cure, and consequences [11,12]. Our patients strongly identified with the label of CLD, with many expressing relief to have a diagnosis to identify with their symptoms. Furthermore, attributing symptoms to active borreliosis, whether or not objective testing supported this diagnosis, was empowering in the sense that treatments (prolonged antibiotic courses and CAM therapies) can be directed towards the bacteria to control and possibly cure the condition. In our sample, the timeline was necessarily chronic (and often cyclical), as we selected patients identifying with CLD and not necessarily having a history of acute Lyme disease. The consequences of CLD in our cohort were remarkable, impacting social relationships, and activities of daily living, as well as perceptions of mortality and debility.

Some literature has classified CLD as ‘medically unexplained symptoms’ [1]. Symptomatically, CLD can resemble other such conditions including chronic fatigue syndrome and fibromyalgia [21], as well as neurologic conditions such as amyotrophic lateral sclerosis [22] or multiple sclerosis [23]. The lack of clear pathophysiology often results in extensive diagnostic workups and significant iatrogenic complications [24]. Like many chronic pain conditions, depressive and anxiety disorders are prevalent and can be overlooked [19]. At least 13% of outpatient visits can be attributed to medically unexplained symptoms [24].

In one study, a majority of primary care physicians described their attitudes towards patients as negative and dismissing [25] while another study found substantial discordance between patient and physician treatment goals [26]. Previous studies of other medically unexplained conditions note that patients often identify as ‘medical orphans’ without a medical specialty home to seek treatment. Further, their symptoms are frequently labeled psychosomatic, and their stories described as ‘chaos narratives’ lacking a defined illness trajectory with uncertainty about persistence or improvement of symptoms [27].

Our participants reported similar experiences. Congruent with our findings, Nunes et al. [28] also found patient concerns regarding about the illness severity to predominate over concerns about symptomatic relief in a qualitative study of patients with medically unexplained symptoms. In a study of persons with fibromyalgia, LaChapelle et al. found that patients reported mixed feelings about support groups; some valued the ‘safe haven’ aspect as an encouraging environment with peers, while other patients reported discouragement from others, perceived to be "building their whole life around the disease" [29].

As is true of many health conditions that are poorly understood and often resistant to conventional treatments, medically unexplained conditions often compels those afflicted to seek CAM therapies and providers [30,31] including over-the-counter products, mind-body practices, dietary modifications, acupuncture, and chiropractic [32].

The majority (11/12) of our participants used one or more forms of CAM, and most consulted a CAM provider. To the best of our knowledge, this is the first study to report frequent CAM use among CLD patients. The high prevalence of CAM was unexpected given that recruitment occurred in general (not CAM-oriented) email lists. It is possible that patients were more open to discussing CAM in a study led by a CAM-trained investigator (AA), whereas patients often feel reluctant to discuss CAM use with conventional providers [33].

It is possible that CLD patients are attracted to unconventional providers – those providing extensive antibiotic regimens beyond current guidelines (‘Lyme-literate medical doctors’) and/or CAM providers due to the lack of symptomatic relief, as well as a perceived lack of sympathy to the diagnosis of CLD in more conventional settings. Our participants reported greater satisfaction with providers described as being ‘open minded’ and supported the use of CAM therapies, while expressing frustration with doctors perceived as dismissive after serology ruled out Lyme disease. The extensive financial expenditures reported may be reflective of a desire for therapeutic options beyond those conventionally available [34].

Some reported CAM therapies may be of questionable value (i.e., the Rife Machine purporting to detect ‘vibrations’ in the body to aid in diagnosis and treatment), while others may be directly harmful. For example, thujone-containing extracts of *Artemesia absinthium* (wormwood) can cause seizures, rhabdomyolysis, and acute renal failure [35], while the use of oral colloidal silver is associated with neurological deficits, diffuse silver deposition in visceral organs, and renal damage. Nevertheless, our participants did not report any adverse effects from CAM therapies.

This study has several limitations. It was restricted to one geographic area where Lyme disease is endemic (Connecticut, USA), and social awareness of CLD is prevalent. Though our sample was small, theoretical saturation was reached. Furthermore, our participants were ethnically homogenous and computer literate, and *a priori*, identified with CLD. Our results may therefore not apply to more diverse groups of CLD patients and those in other geographic locations.

## Conclusions

This study provides insights into the health beliefs and lived experiences of CLD patients. Most participants experienced a considerable decline in their health status attributed to CLD and expressed concerns about long-term debilitation. Participants reported polarized experiences in healthcare settings and sought out CAM providers and therapies. Many participants sought out and were treated by highly invasive (long-term antibiotic regimens), at strong contrast to other chronic conditions where less-invasive therapies are desired [36].

Patient-centered outcomes research approaches [37], emphasizing research propelled by patient interest, may be most appropriate for medically unexplained conditions such as CLD. Actively engaged and sympathetic clinical encounters may foster greater satisfaction in healthcare settings, where patient concerns are fully acknowledged and addressed. Further study of the perceptions and desires of CLD patients as well as the use, efficacy, and safety of unconventional therapies for CLD are warranted.

## Competing interests

The authors declare that they have no competing interests.

## Authors’ contributions

AA: study design, data collection, data analysis, data interpretation, writing. LV: study design, data analysis, data interpretation, writing. RL: data analysis, data interpretation. TRW: editorial support, writing. ERC: study design, data interpretation, writing. All authors read and approved the final manuscript.

## Pre-publication history

The pre-publication history for this paper can be accessed here:

http://www.biomedcentral.com/1471-2296/15/79/prepub

## References

[B1] Feder HM Jr Johnson BJ O'Connell S Shapiro ED Steere AC Wormser GP Agger WA Artsob H Auwaerter P Dumler JS Bakken JS Bockenstedt LK Green J Dattwyler RJ Munoz J Nadelman RB Schwartz I Draper T McSweegan E Halperin JJ Klempner MS Krause PJ Mead P Morshed M Porwancher R Radolf JD Smith RP Jr Sood S Weinstein A Wong SJ A critical appraisal of "chronic Lyme disease" N Engl J Med 2007 357 14 1422 1430 10.1056/NEJMra072023 17914043

[B2] Wormser GP Dattwyler RJ Shapiro ED Halperin JJ Steere AC Klempner MS Krause PJ Bakken JS Strle F Stanek G Bockenstedt L Fish D Dumler JS Nadelman RB The clinical assessment, treatment, and prevention of lyme disease, human granulocytic anaplasmosis, and babesiosis: clinical practice guidelines by the Infectious Diseases Society of America Clin Infect Dis 2006 43 9 1089 1134 10.1086/508667 17029130

[B3] Cameron D Gaito A Harris N Bach G Bellovin S Bock K Bock S Burrascano J Dickey C Horowitz R Phillips S Meer-Scherrer L Raxlen B Sherr V Smith H Smith P Stricker R ILADS Working Group Evidence-based guidelines for the management of Lyme disease Expert Rev Anti Infect Ther 2004 2 1 Suppl S1 S13 15581390 10.1586/14789072.2.1.s1

[B4] Cooper JD Feder HM Jr Inaccurate information about lyme disease on the internet Pediatr Infect Dis J 2004 23 12 1105 1108 15626946

[B5] Burrascano J Advanced Topics in Lyme Disease 2008 16 http://www.ilads.org/lyme/B_guidelines_12_17_08.pdf

[B6] Baker PJ Perspectives on "chronic Lyme disease" Am J Med 2008 121 7 562 564 10.1016/j.amjmed.2008.02.013 18589049

[B7] Johnson M Feder HM Jr Chronic Lyme disease: a survey of Connecticut primary care physicians J Pediatr 2010 157 6 1025 1029 e1021-1022 10.1016/j.jpeds.2010.06.031 20813379

[B8] Ballantyne C The chronic debate over Lyme disease Nat Med 2008 14 11 1135 1139 18989271 10.1038/nm1108-1135

[B9] Bradley EH Curry LA Devers KJ Qualitative data analysis for health services research: developing taxonomy, themes, and theory Health Serv Res 2007 42 4 1758 1772 10.1111/j.1475-6773.2006.00684.x 17286625 PMC1955280

[B10] Janz NK Becker MH The health belief model: a decade later Health Educ Q 1984 11 1 1 47 10.1177/109019818401100101 6392204

[B11] Heijmans M de Ridder D Assessing illness representations of chronic illness: explorations of their disease-specific nature J Behav Med 1998 21 5 485 503 10.1023/A:1018788427100 9836133

[B12] Leventhal H Meyer D Nerenz D Rachman S The common sense representation of illness danger Medical Psychology, Volume 2 1980 New York: Pergamon 7 30

[B13] Creswell JW Qualitative, Quantitative and Mixed Methods Approaches 2003 Thousand Oaks: Sage Publications

[B14] Lindseth A Norberg A A phenomenological hermeneutical method for researching lived experience Scand J Caring Sci 2004 18 2 145 153 10.1111/j.1471-6712.2004.00258.x 15147477

[B15] Bell IR Cunningham V Caspi O Meek P Ferro L Development and validation of a new global well-being outcomes rating scale for integrative medicine research BMC Complement Altern Med 2004 4 1 10.1186/1472-6882-4-1 14725717 PMC343287

[B16] Bacon RM Kugeler KJ Mead PS Centers for disease C, prevention: surveillance for Lyme disease–United States, 1992–2006 MMWR Surveill Summ 2008 57 10 1 9 18830214

[B17] Klempner MS Hu LT Evans J Schmid CH Johnson GM Trevino RP Norton D Levy L Wall D McCall J Kosinski M Weinstein A Two controlled trials of antibiotic treatment in patients with persistent symptoms and a history of Lyme disease N Engl J Med 2001 345 2 85 92 10.1056/NEJM200107123450202 11450676

[B18] Kugeler KJ Griffith KS Gould LH Kochanek K Delorey MJ Biggerstaff BJ Mead PS A review of death certificates listing lyme disease as a cause of death in the United States Clin Infect Dis 2010 52 2 364 367 21189272 10.1093/cid/ciq157

[B19] Hassett AL Radvanski DC Buyske S Savage SV Sigal LH Psychiatric comorbidity and other psychological factors in patients with "chronic Lyme disease" Am J Med 2009 122 9 843 850 10.1016/j.amjmed.2009.02.022 19699380 PMC2751626

[B20] Ljostad U Mygland A The phenomenon of ‘chronic Lyme’; an observational study Eur J Neurol 2012 19 8 1128 1135 10.1111/j.1468-1331.2012.03691.x 22416947

[B21] Shapiro ED Dattwyler R Nadelman RB Wormser GP Response to meta-analysis of Lyme borreliosis symptoms Int J Epidemiol 2005 34 6 1437 1439 author reply 1440–1433 10.1093/ije/dyi241 16319106

[B22] Qureshi M Bedlack RS Cudkowicz ME Lyme disease serology in amyotrophic lateral sclerosis Muscle Nerve 2009 40 4 626 628 10.1002/mus.21438 19697382

[B23] Stricker RB Johnson L ‘Rare’ infections mimicking multiple sclerosis: consider Lyme disease Clin Neurol Neurosurg 2010 113 3 259 260 21168953 10.1016/j.clineuro.2010.11.017

[B24] Ring A Dowrick CF Humphris GM Davies J Salmon P The somatising effect of clinical consultation: what patients and doctors say and do not say when patients present medically unexplained physical symptoms Soc Sci Med 2005 61 7 1505 1515 10.1016/j.socscimed.2005.03.014 15922499

[B25] Salmon P Peters S Clifford R Iredale W Gask L Rogers A Dowrick C Hughes J Morriss R Why do general practitioners decline training to improve management of medically unexplained symptoms? J Gen Intern Med 2007 22 5 565 571 10.1007/s11606-006-0094-z 17443362 PMC1855690

[B26] Nordin TA Hartz AJ Noyes R Jr Anderson MC Rosenbaum ME James PA Ely JW Agarwal N Levy BT Empirically identified goals for the management of unexplained symptoms Fam Med 2006 38 7 476 482 16823672

[B27] Nettleton S Watt I O’Malley L Duffey P Understanding the narratives of people who live with medically unexplained illness Patient Educ Couns 2005 56 2 205 210 10.1016/j.pec.2004.02.010 15653250

[B28] Nunes J Ventura T Encarnacao R Pinto PR Santos I What do patients with medically unexplained physical symptoms (MUPS) think? A qualitative study Ment Health Fam Med 2013 10 2 67 79 24427173 PMC3822638

[B29] Lachapelle DL Lavoie S Boudreau A The meaning and process of pain acceptance. Perceptions of women living with arthritis and fibromyalgia Pain Res Manag 2008 13 3 201 210 18592056 10.1155/2008/258542 PMC2671308

[B30] Pioro-Boisset M Esdaile JM Fitzcharles MA Alternative medicine use in fibromyalgia syndrome Arthritis Care Res 1996 9 1 13 17 10.1002/art.1790090105 8945108

[B31] Paterson C Taylor RS Griffiths P Britten N Rugg S Bridges J McCallum B Kite G team Cs Acupuncture for ‘frequent attenders’ with medically unexplained symptoms: a randomised controlled trial (CACTUS study) Br J Gen Pract 2011 61 587 e295 e305 10.3399/bjgp11X572689 21801508 PMC3103692

[B32] Dimmock S Troughton P Bird H Factors predisposing to the resort of complementary therapies in patients with fibromyalgia Clin Rheumatol 1996 15 5 478 482 10.1007/BF02229645 8894361

[B33] Tasaki K Maskarinec G Shumay DM Tatsumura Y Kakai H Communication between physicians and cancer patients about complementary and alternative medicine: exploring patients’ perspectives Psychooncology 2002 11 3 212 220 10.1002/pon.552 12112481

[B34] Johnson L Aylward A Stricker RB Healthcare access and burden of care for patients with Lyme disease: a large United States survey Health Policy 2011 102 1 64 71 10.1016/j.healthpol.2011.05.007 21676482

[B35] Wormwood Natural Medicines Comprehensive Database [database on the internet] 2012 Stockton, CA: Therapeutic Research Faculty Available from http://www.naturaldatabase.com

[B36] Ali A Rakel D Lyme Disease Integrative Medicine 2012 3 Philadelphia, PA: Elsevier Medical Publishing

[B37] Krumholz HM Selby JV Patient-Centered Outcomes Research I: Seeing through the eyes of patients: the Patient-Centered Outcomes Research Institute Funding Announcements Ann Intern Med 2012 157 6 446 447 22847044 10.7326/0003-4819-157-6-201209180-00519

